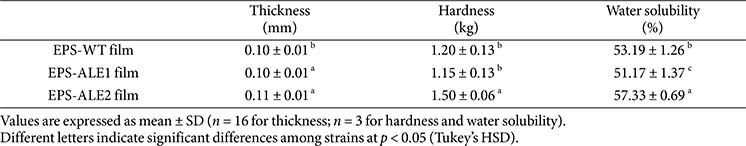# Hydrodynamic Size Modulation of Exopolysaccharides from *Leuconostoc mesenteroides* WiKim33 via Genetic Modification and Its Impact on the Properties in Film

**DOI:** 10.4014/jmb.2508.08008

**Published:** 2025-11-27

**Authors:** Yeong Yeol Kim, Jong-Cheol Kim, Namhee Kim, Seul-Gi Jeong, Jung Eun Yang, Chang Hee Jeong, Ho Myeong Kim, Yong-Su Song, Woo-Jin Jung, Hae Woong Park

**Affiliations:** 1 Advanced Convergence Research Division, World Institute of Kimchi, Gwangju 61755, Republic of Korea; 2 Department of Integrative Food, Bioscience and Biotechnology, Chonnam National University, Gwangju 61186, Republic of Korea; 3 Institute of Environmentally-Friendly Agriculture (IEFA), Chonnam National University, Gwangju 61755, Republic of Korea; 4 Department of Agricultural and Biological Chemistry, College of Agricultural and Life Sciences, Chonnam National University, Gwangju 61755, Republic of Korea

**Keywords:** Lactic acid bacteria, adaptive laboratory evolution, exopolysaccharide, macromolecule, dextran

## Abstract

This study explored the effects of genetic modifications on exopolysaccharide (EPS) of *Leuconostoc mesenteroides* WiKim33, which was subjected to heterotypic shock-induced adaptive laboratory evolution (ALE). We examined three strains: wild-type, ALE1, and ALE2. Among these strains, ALE1 produced the highest EPS (3.87 g/l), while ALE2 produced the lowest (3.27 g/l). Nuclear magnetic resonance analysis revealed that mutations affected the hydrodynamic diameter of EPS. The ALE1 strain had the smallest EPS (11.15 nm), while ALE2 produced the largest EPS (43.27 nm). These differences in hydrodynamic size significantly affected the physical properties of EPS films. Films incorporating EPS from ALE1 exhibited reduced water solubility and hardness compared with the film containing EPS from ALE2. Confocal laser-scanning microscopy revealed that a larger EPS hydrodynamic size induced phase separation and weakened the structural integrity of the film. The current findings highlight ALE to alter the hydrodynamic size of EPS and understand its physical properties within the resulting film.

## Introduction

Lactic acid bacteria (LAB) are extensively utilized across various industries, including pharmaceuticals, fermented food and beverage production, and functional cosmetics. With the growing demand for LAB, there is an anticipated increase in industrial fermented waste [1]. The uncontrolled disposal of such waste raises significant environmental concerns, underscoring the need for effective valorization strategies. Among different types of waste, spent media from LAB culture presents a promising opportunity for valorization through the production of valuable polysaccharides. LAB is known to produce two types of EPS: heteropolysaccharides (HePS) and homopolysaccharides (HoPS). HePS synthesis involves a multi-step intracellular process, which includes sugar uptake, nucleotide sugar biosynthesis, polymerization, and subsequent EPS transport [2]. In contrast, HoPS synthesis is catalyzed by extracellular or cell wall-associated enzymes, such as dextransucrase, which directly utilize sucrose as a substrate at the cell surface. This process bypasses the need for extensive intracellular uptake and conversion steps but still depends on the availability of extracellular sucrose [3]. Following bacterial metabolism during cultivation, the media contain abundant EPS produced by the bacteria in response to environmental stresses such as salinity, drought, acidity, heat, and osmotic pressure [4].

EPS has garnered substantial interest due to its unique chemical and structural properties, making it valuable for the food and non-food industries. EPS serves as a viscosity enhancer, texture modifier, and emulsifying agent while providing health benefits, including immunomodulatory, anti-infectious, and anti-inflammatory effects [5]. In response to industrial needs, research on EPS production has primarily focused on optimizing yield and modulating EPS properties under controlled conditions by varying factors such as inoculum volume, temperature, pH, nutrient sources, fermentation time, and agitation speed [5, 6]. These strategies have effectively produced EPS with diverse molecular weights and quantities. In our previous research, we successfully demonstrated the potential of spent media from *Leuconostoc mesenteroides* culture to produce non-toxic natural antioxidative EPS, addressing the increase in waste generated by the demand for fermented products [7].

Currently, we are exploring the application of adaptive laboratory evolution (ALE) to develop desirable strains with improved EPS production capacities. ALE offers a promising approach to investigating the effects of genetic mutation on EPS production and structural properties while avoiding the safety concerns associated with artificial genetic engineering tools. ALE is a well-established method of biological mutagenesis and adaptation employed to develop desirable characteristics in controlled environments [8]. Numerous studies have highlighted the mutagenesis-induced development of favorable properties, such as stress tolerance, increased product yield, metabolic functional differences, genome size variations, and differential gene expression levels, without artificial genetic modification [9]. Natural mutagenesis and adaptation can mitigate off-target mutations and the potential genome damage associated with artificial mutants, presenting a safer alternative for industrial applications [10, 11]. Furthermore, insights into mutagenic conditions can provide valuable information on the potential of convergent evolution, characterized by the development of analogous phenotypic traits that share form or function despite species differences in response to environmental adaptation [12, 13].

Our previous study reported the development of a lyophilization-resistant strain exhibiting a 331% increase in membrane EPS thickness following continuous incubation after exposure to heterotypic shocks [9]. The EPS thickness was closely related to bacterial viability after the freeze-drying process, depending on different membrane matrices. Therefore, it is important to understand the structural characteristics of EPS induced by heterotypic shocks for future ALE strain development. The primary objective of the current study was to investigate the genetic mutations in *Leu. mesenteroides* WiKim33 after heterotypic shock adaptation, and assess how these mutations affect EPS productivity and hydrodynamic size differences. Additionally, we aimed to evaluate the modified hydrodynamic size of EPS as a film component to understand its physical properties through environmental stress adaptation.

## Materials and Methods

### Microorganisms

Wild-type *Leu. mesenteroides* WiKim33 (KFCC 11640P, WT) obtained from kimchi and two mutants of ALE1 (reported as a lyophilization-resistant strain named *Leu. mesenteroides* WiKim0205) and ALE2 (selected mutant as a negative control) derived from our previous study were used in the current study [9]. For the development of mutant strains, the ALE was performed for 50 cycles. *Leu. mesenteroides* WiKim33 WT were subjected to combined heat and osmotic pressure stress. The WT cells were cultured in sterilized MRS at 30°C for 22 h, harvested by centrifugation (14,308 ×*g*, 15 min, 25°C), and re-suspended in the same volume of 60% (v/v) glycerol solution to induce osmotic shock. For heat treatment, the resulting suspension was placed in a thermally controlled water bath at 40°C. After 1 h of heterotypic shock via combined heat and osmotic stress, the cell suspension was serially diluted, spread onto MRS agar plates, and then incubated at 30°C for 24 h. Eleven colonies were randomly selected from the 50th cycles. After freeze-drying process, two mutant strains (*i.e.*, the highest and the lowest freeze-drying tolerance) were selected for this study. The bacterial strains were cultured in De Man, Rogosa, and Sharpe (MRS) medium (Difco Laboratories, USA) after sterilization at 30°C for 24 h and were stored at –80°C (MDF4V; Panasonic, Japan) with 17% glycerol.

### Chemicals

To produce EPS, MRS without glucose was purchased from KisanBio (Republic of Korea), and sucrose was purchased from Samyang Genex (Republic of Korea). For the purification of EPS, 95.0% ethanol was purchased from Samchun Chemical Co. (Republic of Korea), and trichloroacetic acid was purchased from Wako Pure Chemical Co. (Japan). To prepare the edible film, gelatin from bovine skin and ProClin 300 were purchased from Merck (Germany), and glycerol was purchased from Duchefa Biochemie (Netherlands). To stain gelatin for structural observation after film manufacturing, rhodamine B was purchased from Merck.

### DNA Isolation and Whole-Genome Re-Sequencing of Mutants

Genomic DNA from the two mutants was re-sequenced for comparison with the WT. ALE1 and ALE2 were plated on MRS agar media, and a single colony from each plate was isolated. Genomic DNA was extracted using a genomic DNA preparation kit (Qiagen, Germany), followed by library construction. DNA libraries were constructed using the TruSeq Nano DNA kit (Illumina, USA). The extracted DNA was randomly fragmented and repaired to blunt ends using an end repair enzyme included in the kit. The genomes were sequenced using *Leu. mesenteroides* WiKim33 WT as the reference genome (GenBank accession number GCA_003433375.1) with the BWA program to produce aligned reads. Duplicate reads were removed with Picard, and variant calling was performed using SAMtools after mapping.

### Preparation of EPSs

The purified EPS was obtained following our previous study [7]. The three strains of *Leu. mesenteroides* WiKim33 (WT, ALE1, and ALE2) were cultured in MRS containing 5% sucrose and fermented at 30°C for 24 h to produce EPS after incubation in sterilized MRS broth. The supernatant of cultured media was obtained after being heated at 100°C for 10 min in a water bath for enzymatic inactivation and centrifugation (14,308 ×*g*, 10 min). Crude EPSs were obtained by adding three times the volume of 95.0% ethanol to the supernatant and storage at 4°C overnight and centrifugation. The obtained pellets from each step were dried at 90°C for 30 min to reduce residual ethanol. Crude EPSs were deproteinized with 4% trichloroacetic acid, and deproteinized EPSs were obtained using the same ethanol precipitation method. The deproteinized EPSs were dialyzed using Slide-A-Lyzer Dialysis Cassettes (10K MWCO, Thermo Fisher scientific, USA) for three days, with daily water changes. After dialysis, EPS solutions were collected in a centrifuge tube and freeze-dried to obtain powdered EPSs. EPS production for each strain was conducted in triplicate to ensure conversion rate and productivity. The purified EPSs were named EPS-WT (EPS isolated from WT), EPS-ALE1 (EPS isolated from ALE1), and EPS-ALE2 (EPS isolated from ALE2).

### Structural Analysis of EPSs

To obtain the chemical structure of each EPS, 15 mg of EPS was dissolved in 0.5 ml of D_2_O and filtered for sample preparation. ^1^H, ^13^C, and 2D (HSQC) nuclear magnetic resonance (NMR) experiments were performed using an AVANCE III 600 MHz spectrometer (Bruker, USA). The 2D (COSY and NOESY) NMR experiments were conducted with an AVANCE III 500 MHz spectrometer. The 2D ^1^H-^13^C heteronuclear single quantum correlated (HSQC) spectra with a data matrix composed of 1,024×128, 2D ^1^H-^1^H correlation spectroscopy (COSY) spectrum with a data matrix composed of 2,048×128, and 2D ^1^H-^1^H nuclear-overhauled effect spectroscopy (NOESY) spectrum 2,048×160 were obtained for structural identification of purified EPSs.

### Hydrodynamic Diameter Measurement

The hydrodynamic diameter of each EPS was measured using dynamic light scattering with Zetasizer Nano ZSP (Malvern Panalytical, Ltd., Malvern UK) at 25°C. The 15 mg of EPS were dissolved in H2O and filtered through a 0.22 μm filter to prepare the sample for hydrodynamic diameter measurement.

### Molecular Weight Analysis

The molecular weight of EPS was determined using a multi-angle light scattering (MALS) system (DAWN Heleos II, Wyatt Technology, USA) equipped with an Optilab T-rEX refractive index (RI) detector and controlled via ASTRA 6 software. The system was connected to a Shimadzu HPLC operating under LS-RI mode at 25°C. Sample analysis was carried out using a TSK-gel GMPWXL column (Tosoh Bioscience, Japan) with a flow rate of 0.5 ml/min and an injection volume of 100 μl. The molecular weight (MW), number-average molecular weight (Mn), and polydispersity index (PDI) were calculated from light scattering and RI signals based on the Zimm model.

### Surface Architectural Investigation

To investigate the architectural differences between EPS, the Apreo 2 S LoVac (Thermo Fisher Scientific) was used. Each EPS was placed on the stub with conductive carbon tape. The beam voltage was varied to minimize sample charging while maximizing resolution.

### EPS Film Preparation

The EPS film was prepared following the study of gelatin-dextran film with slight modification [13]. The EPS and gelatin mixed solution was prepared by dispersing EPS at 4% (w/v) and gelatin at 3% (w/v) in purified water containing 1.4% (w/v) glycerol as a plasticizer. ProClin 300 was added at 0.001% (v/v) to the solution to prevent microbial growth. The film forming solution was adjusted to pH 7.0. The films were prepared using a modified gelatin drying method. Film-forming solutions of 5 g were poured into Petri dishes and dried in a desiccator at 25°C for 4 days. After drying, the films were peeled off and conditioned under the same conditions for 3 days before the experiments.

### Comparative Visual Observation of EPS Films

The appearance of the prepared EPS film without staining was recorded using a mobile phone camera (Samsung Galaxy S23). The turbidity was compared using a paper with the “World Institute of Kimchi” logo (https://www.wikim.re.kr).

### Fluorescence 3D Image Stacking of EPS Film Using Confocal Laser-Scanning Microscopy

The films stained with rhodamine B were placed on a glass slide and observed using a confocal microscope (LSM700, Carl Zeiss, Germany). The excitation wavelength was 552 nm, and the objective lens was 20× (with 2×digital zoom). The 3D images were constructed from traditional 2D images. All image files were obtained at 1,024×1,024 pixels in TIFF format [14, 15].

### Film Properties Measurement

Film thickness was measured using a caliper with an accuracy of ± 0.01 mm. The thickness of each EPS film sample was measured at 16 random points, whereafter the average and standard deviation were calculated. The hardness profile was obtained through a penetration test with a texture analyser (CT-3; Brookfield Engineering Laboratories, Inc., USA) equipped with a 2 mm flat-ended cylindrical probe (TA39). The measurement was performed at a pretest at 2.0 mm/s, test at 0.5 mm/s, and return at 5 mm/s. The greatest force of the force-time curve was selected as the firmness. All measurements were performed in triplicates, with the hardness (kg) expressed as the mean and standard deviation [16]. To compare the difference in solubility between the EPS films, the prepared films were cut into 15 mm × 15 mm pieces and dried to a constant weight in an oven (105°C). After obtaining a constant weight, the films were immersed in 200 ml of distilled water at 25°C for a day and then dried in an oven to obtain a constant weight. The water solubility was calculated as follows:

$$\text{Water solubility (\%)}=1-\frac{W0-W1}{W0}\times 100\%$$

W0: the constant weight of the film before immersion in distilled water

W1: the constant weight after immersion and drying.

### Statistical Analysis

All data are expressed as the average of triplicate experiments. SPSS (version 20; IBM, USA) was used for statistical analysis. Analysis of variance followed by Tukey’s post hoc test was used to determine significant differences between samples at *P* < 0.05.

## Results and Discussion

### Mutant Isolation and Genetic Modification via Heterotypic Shock

Previously, we randomly isolated mutants following heterotypic shock adaptation and assessed their freeze-drying tolerance [9]. Two mutants, ALE1 and ALE2, were selected based on their viability after freeze-drying, with ALE1 showing the highest and ALE2 the lowest viability among the mutants (Supplementary Data S1). Significant differences among strains were observed in both DW (*F* = 125.33, *df* = 2, 12, *p* < 0.001) and trehalose (*F* = 187.41, *df* = 2, 12, *p* < 0.001).

We analyzed the genetic variations among three strains of *Leu. mesenteroides* WiKim33: WT, ALE1, and ALE2 (Table 1). Genetic modifications in these strains, which arose during adaptive evolution, were identified through whole-genome resequencing. This analysis uncovered three and seven single-nucleotide polymorphisms (SNPs) in the coding DNA sequences of ALE1 and ALE2, respectively, when compared to the WT strain. Additionally, two and three SNPs were identified in the promoter regions of ALE1 and ALE2. These SNPs represent adaptations resulting from repeated heterotypic shock exposure.

Microorganisms subjected to stressful environments adjust their gene expression to enhance stress tolerance. Successive stress exposures lead to the accumulation of differentially expressed genes (DEGs) over time. Our prior studies indicated that heterotypic shock-induced mutants exhibited DEGs linked to increased stress resistance through enhanced cellular encapsulation [9]. Consistent with these findings, genetic mutations primarily related to bacterial EPS synthesis and cellular encapsulation were identified, suggesting that these mutant genes modulate the encapsulating matrix, particularly influencing EPS production (Supplementary Data S2).

### Productivity of EPS from Waste Media

The highest EPS production was observed in ALE1 at 3.87 g/l, followed by WT at 3.34 g/l and ALE2 at 3.27 g/l in sugar-enriched media (*F* = 22.76, *df* = 2,6, *p* = 0.002) (Table 2). This EPS production result correlated with freeze-drying tolerance (Supplementary Data S1). In our previous study, *Leu. mesenteroides* WiKim32 exhibited increased EPS matrix thickness in response to environmental stress, with the produced EPS matrix serving as a protective agent [16]. A correlation between EPS productivity and stress resistance was noted in the isolated mutants. Differences in genome and EPS productivity motivate a structural comparison of the purified EPS from each strain.

### Structural Analysis of EPS from Media Waste

**Fig. 1:**
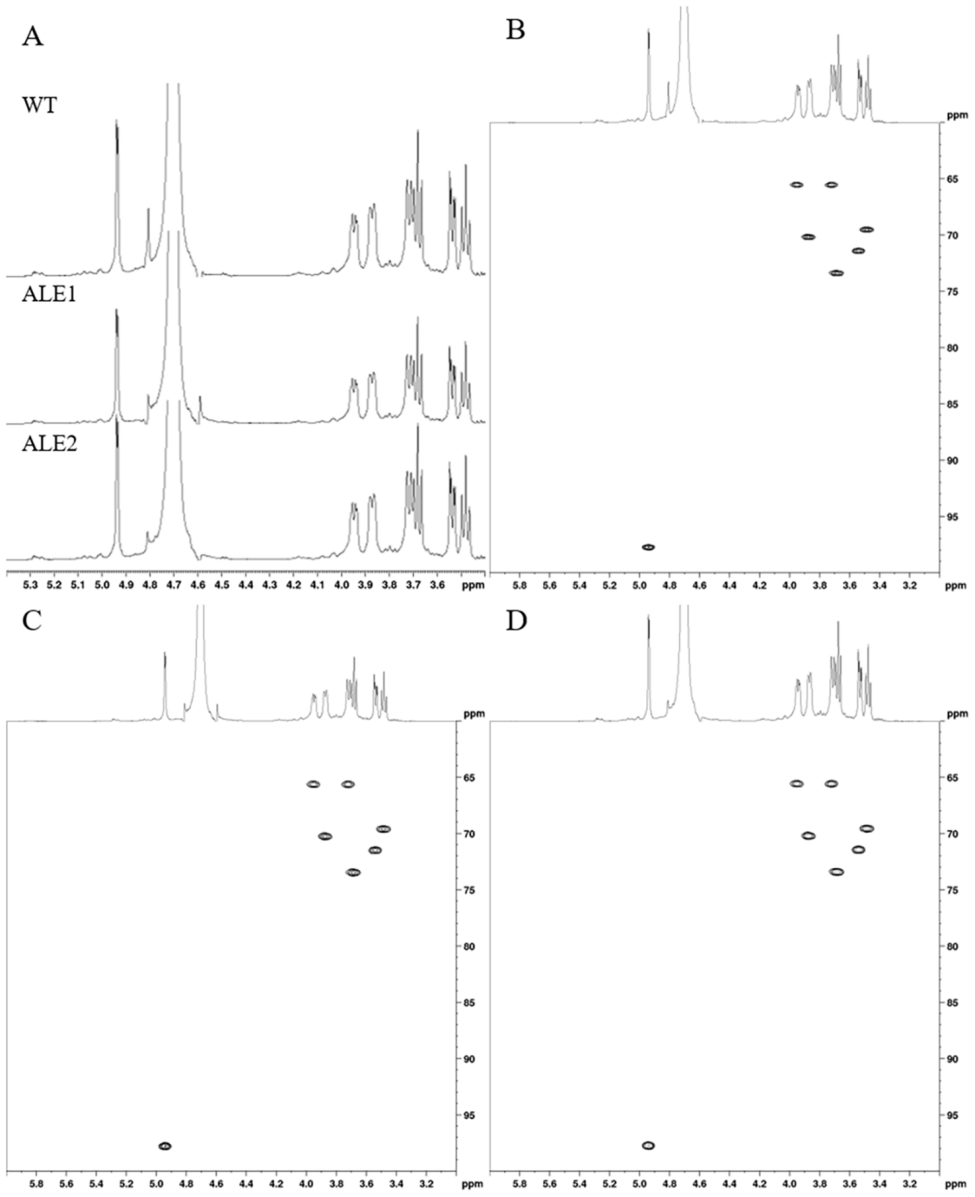
Structural characterization of exopolysaccharides from *Leuconostoc mesenteroides* WiKim33 and its mutant strains. (**A**) ^1^H NMR spectra EPSs; (**B-D**) 2D HSQC NMR spectra of EPSs purified from WT, ALE1, and ALE2 waste media.

**Fig. 2:**
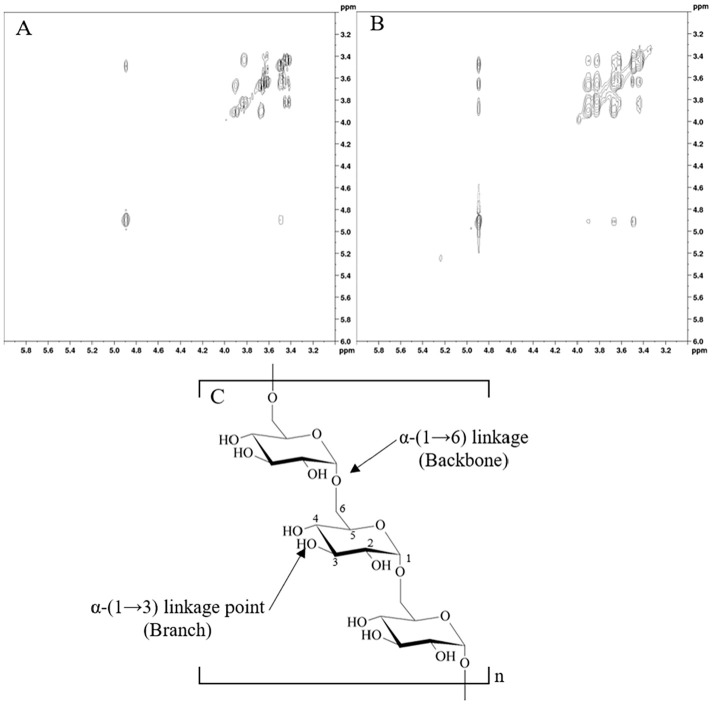
Structural analysis of exopolysaccharides (EPS) from *Leuconostoc mesenteroides* using 2D NMR. (**A**) COSY spectrum of proton-proton scalar couplings; (**B**) NOESY spectrum of spatial proton-proton proximities; (**C**) Proposed structural representation.

Structural analysis experiments were conducted using a 600 MHz NMR spectrometer with D_2_O as the solvent at 298 K. The structural characterization of purified EPS from each strain was performed using ^1^H, ^13^C, and 2D NMR spectroscopy (Figs. 1, 2, and Supplementary Data S3). The NMR spectra revealed that the EPSs shared a common chemical structure [7]. The ^1^H NMR spectra for each EPS are shown in Fig. 1A. The ^1^H NMR analysis displayed chemical shifts consistent with shared spectra for the EPSs produced by WT, ALE1, and ALE2 strains, respectively. An anomeric signal at 4.93 and 5.27 ppm, typical glycosidic linkage of α-(1→6) and α-(1→3), were shared in all EPS samples which means the presence of backbone and branch chain. The results align proton signal previously reported for dextran produced by the *Leu. mesenteroides* strain [18]. The results of ^13^C NMR analysis strongly suggested that the EPSs are composed of a single exopolysaccharide, most likely dextran (Supplementary Data S3). In addition, a heteronuclear 2D NMR experiment, specifically HSQC NMR, was conducted (Fig. 1B-1D and Supplementary Data S4). The results showed a correlation between H-1/C-1 (4.93 ppm in ^1^H NMR and 97.74 ppm in ^13^C NMR) and two negative H6 intensities (3.69 and 3.96 ppm in ^1^H NMR and 65.57 ppm in ^13^C NMR), confirming structural homology among the EPS samples. This analysis verified the structural similarity of the EPS, even in cases where the strain underwent genetic mutation due to environmental stress, as demonstrated by correlated chemical shifts.

Homo- and hetero-nuclear 2D NMR experiments were performed to assign all spin systems and determine the specific saccharide sequence. The anomeric configuration of each monosaccharide unit was assigned based on the ^3^J_H-1, H-2_ coupling constant values obtained from COSY analysis, while the vicinal ^3^J_H, H_ ring coupling constants allowed for the identification of the relative configuration of each sugar (Fig. 2A).

In the COSY spectrum, proton H1 (4.89 ppm) was identified as the starting point for sequentially assigning protons along the pyranoside backbone. By tracing the spin system, H2 (3.48 ppm) and H3 (3.62 ppm) were assigned, followed by H4 (3.43 ppm), H5 (3.82 ppm), and H6 (3.66 and 3.90 ppm) step-by-step, based on the scalar coupling correlations observed in the COSY and HSQC spectra. These results confirmed the pyranoside ring structure [18]. The corresponding carbon C1 was readily identified by a strong one-bond correlation between H1 and C1 in the HSQC spectrum. Further correlations between other protons and carbons (*e.g.*, H2-C2, H3-C3) validated the monosaccharide structure [19].

Spin system-A was identified as a *gluco*-configured sugar residue, as indicated by the axial-axial ^3^J_H, H_ ring coupling constants and the chemical shift values, consistent with the *gluco*-configuration of pyranose rings in a ^4^C_1_ conformation. The α-configuration was assigned based on the axial-equatorial ^3^J_H1, H2_ coupling (3.35 Hz)(Fig. 2B). Analysis of scalar correlations in the 2D NMR spectra enabled identification of the exopolysaccharide.

The NOESY were analyzed to investigate spatial proximities between protons within the monosaccharide unit. A strong *intra*-residual NOE contact was observed between H1 and H2, with a calculated distance of ~2.37 Å. This distance was derived from the NOE intensity ratio, using H1-H3 as a reference with a distance of 2.5 Å. These findings provided strong evidence for the overall spatial geometry and rigidity of the pyranoside monosaccharide. Specifically, the short distances of ~2.37 Å (H1-H2) and ~2.5 Å (H1-H3) are characteristic of the ^4^C_1_ chair conformation, where the spatial proximities of protons align with the assign of the dextran-like polysaccharide, composed of a homopolymer of α-(1→6)-Glc [20].

By applying COSY and NOESY, the structure of the exopolysaccharide was determined as a dextran-like homopolymer composed of α-(1→6) linked glucose units. The COSY analysis allowed the sequential assignment of protons along the pyranoside backbone and confirmed the *gluco*-configuration and α-anomeric form. The NOESY analysis provided additional spatial constraints, confirming the ^4^C_1_ chair conformation and overall rigidity of the pyranoside ring. Thus, the combined homo-nuclear 2D NMR analyses effectively elucidated the structural composition, anomeric configuration, glycosylation site, and spatial geometry of the exopolysaccharide.

### Hydrodynamic Diameter Measurement of EPS

**Fig. 3:**
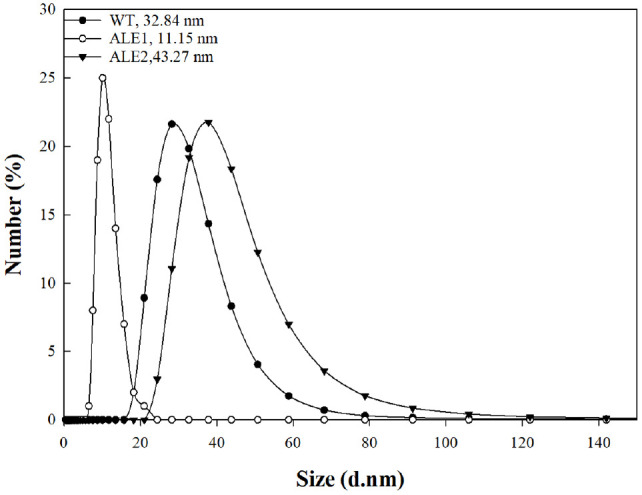
Hydrodynamic diameter measurement of EPS from *Leuconostoc mesenteroides* WiKim33 and mutants.

Dynamic light scattering (DLS) was used to compare the hydrodynamic diameter of EPS in water at a dilute concentration. The results of DLS measurements showed differences in diameter among EPS samples (Fig. 3). The mean hydrodynamic diameters were 32.84 nm for WT-EPS, 11.15 nm for ALE1-EPS, and 43.27 nm for ALE2-EPS, indicating substantial conformational variation among the polymers.

A comparison of the molecular size with MALS analysis revealed that the weight-average MW of the three EPSs was within the same order of magnitude of 7.0 × 10^6^ g/mol, suggesting that the substantial difference in hydrodynamic diameter was not attributable to molecular mass but rather to conformational differences in polymer chain aggregation state (Supplementary Data S5). The more compact and aggregated structure of ALE1-EPS resulted in a smaller apparent hydrodynamic volume, whereas ALE2-EPS exhibited an extended and hydrated coil conformation, producing the largest hydrodynamic diameters among the samples. The hydrodynamic diameter of polymers is highly dependent on their branching architecture rather than solely on MW. A higher degree of branching or shorter arm length induces chain compaction, leading to a smaller hydrodynamic diameter even at identical MW. This theoretical relationship explains the distinct hydrodynamic diameter differences among the EPSs analyzed in this study, despite their similar MW [21].

### Surface Architectural Feature Investigation

**Fig. 4:**
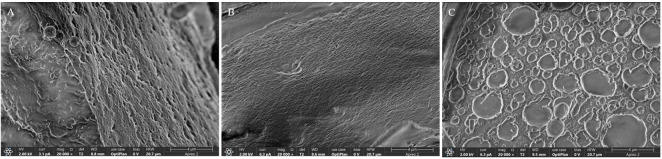
Surface architectural comparison of EPS from *Leuconostoc mesenteroides* WiKim33 and mutants.

Surface characterization of the EPS was performed to examine architectural variations associated with differences in hydrodynamic radius (Fig. 4). Although the EPS samples exhibited comparable molecular weights, variations in hydrodynamic radius likely reflected differences in chain hydration and conformational expansion. Greater hydration within the polymer matrix may have led to the formation of water-enriched microdomains that partially collapsed during solvent removal, producing crater-like surface features. Conversely, more compact and less hydrated structures appeared to undergo uniform shrinkage, resulting in smoother surfaces. These results suggest that the degree of hydration—rather than molecular weight alone—plays a critical role in determining surface irregularities during the drying process. Among the samples, the EPS produced by ALE1, which possessed the smallest hydrodynamic radius, exhibited the most homogeneous and smooth surface morphology, followed by the WT and ALE2, with the latter displaying the greatest surface heterogeneity.

### Optical and Confocal Laser-Scanning Microscopy Observation of EPS Film Appearance

**Fig. 5:**
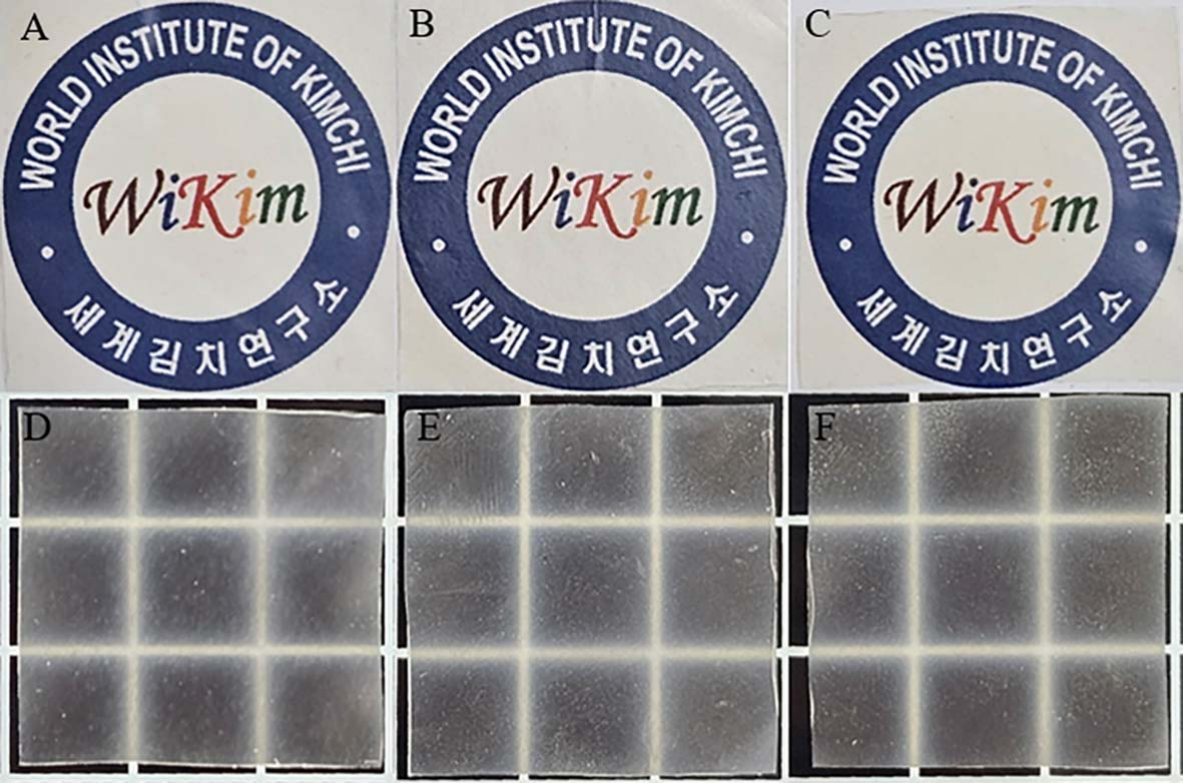
Optical observation of gelatin-EPS film. (**A**) EPS-WT film, (**B**) EPS-ALE1 film, (**C**) EPS-ALE2 film. GE: Gelatin-EPS combined film.

**Fig. 6:**
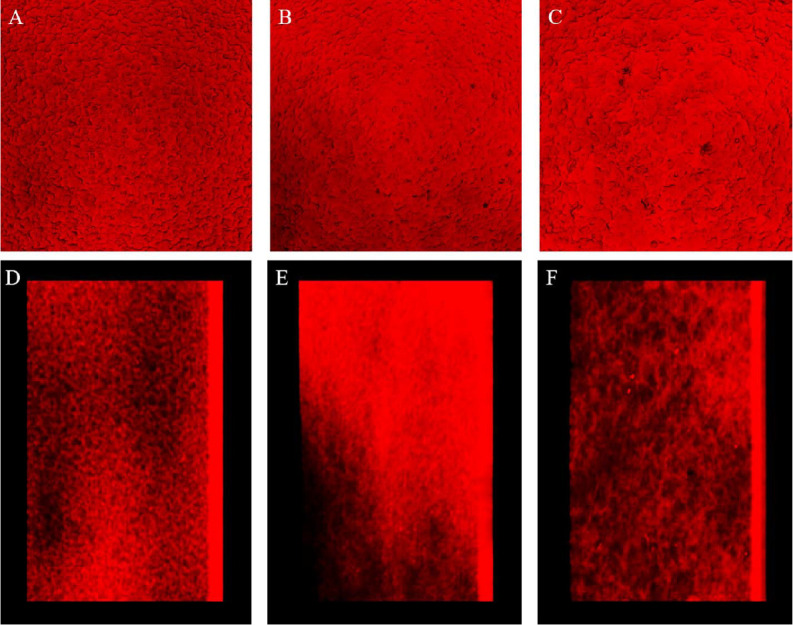
Confocal microscopy observation of gelatin-EPS film. (**A-C**) Topographic image of EPS-WT, EPS-ALE1, and EPS-ALE2 film, (**D-F**) Microstructure observation of EPS-WT, EPS-ALE1, and EPS-ALE2 film.

The optical appearance of EPS films exhibited a smooth and homogenous surface (Fig. 5). However, confocal laser-scanning microscopy provided deeper insight into the film’s microstructure. Using rhodamine B to label gelatin (Fig. 6), we observed varying degrees of phase separation within the EPS matrix, depending on the hydrodynamic size of the incorporated EPS. Higher hydrodynamic size EPS led to increased phase separation and gelatin. aggregation (Supplementary Data S6). Larger EPS molecules occupied more volume, disrupting the homogeneity of the film matrix and leading to larger pore sizes, as seen in the CLSM images. These findings align with previous studies, where macromolecular aggregation increases with hydrodynamic size, promoting phase separation [22]. In contrast, lower hydrodynamic size EPS was more evenly distributed within the film matrix, resulting in a smoother, more integrated structure. In the current study, the hydrodynamic size of EPS presented a role in determining the microstructural organization of EPS film and its resulting properties.

In the case of gelatin/hydroxymethyl cellulose films, no microscopic phase separation is observed at pH 4.5, while gelatin undergoes self-aggregation as the pH increases to 8.6 [23]. Gelatin behaves similarly to polysaccharides by forming a thermoreversible gel state. When gelatin and polysaccharides are kept in a co-soluble state above the gelation temperature, they disperse randomly in solution. Upon cooling, gelatin can either form micro-networks with polysaccharides or undergo phase separation [24]. In this study, the film matrix was intentionally formulated with self-aggregated gelatin in a neutral environment, incorporating different sizes of macromolecular EPS to visualize the effects of hydrodynamic size on film properties.

Proteins and polysaccharides like EPS can form films with distinct mechanical properties. However, their hydrophilic nature often results in poor water barrier properties [25, 26]. Differences in the hydrodynamic size of EPS influence the mechanical behavior, transparency, and optical characteristics of the film. As the hydrodynamic size increases, phase separation becomes more pronounced, potentially affecting the visual clarity and smoothness of the film [14].

### Comparison of Film Properties of EPS Films

To compare the properties of the EPS films, film thickness, hardness, and water solubility were measured (Table 3). Film thickness was influenced by gelatin self-aggregation and phase separation, particularly at higher EPS concentrations and near pH 8.6. Typically, thickness correlated positively with hardness; for instance, the EPS-ALE2 film exhibited both greater thickness and hardness than the EPS-WT and EPS-ALE1 films, attributable to more pronounced phase separation and a denser gelatin-rich phase. The concurrent increase in water solubility and hardness can be explained by the phase-separation behavior of protein–polysaccharide systems. Thermodynamic incompatibility between gelatin and polysaccharides forms biphasic structures of protein- and polysaccharide-rich domains that govern both mechanical strength and water permeability. Incorporation of larger EPS densifies the protein-rich phase, enhancing hardness, while hydrophilic polysaccharide regions create water-permeable pathways that increase solubility despite improved rigidity [14, 25].

In this study, we further observed that even at similar molecular weights, differences in hydrodynamic size resulted in distinct phase-separation patterns. Smaller and compact hydrodynamic diameter of EPS resulted in micro-phase separation with finer and more homogeneous domains, whereas larger hydrodynamic diameter EPS produced coarser and more porous structures within the gelatin network. These micro- and macro-scale separations accounted for the differences in water solubility and mechanical hardness observed among the EPS films. Consistent with this mechanism, larger hydrodynamic-diameter EPS induced more extensive phase separation and pore formation (Fig. 6), resulting in the highest solubility (57.3%) and hardness in the EPS-ALE2 film, whereas the smaller ALE1-EPS produced the lowest solubility (51.2%) and reduced hardness. These findings demonstrate that the hydrodynamic size of EPS has a critical influence on both the extent and scale of phase separation, and consequently on the balance between film strength and water solubility. Moreover, increased gelatin–EPS compatibility suppresses phase separation, leading to reduced water solubility and improved stability. The tunable mechanical and barrier properties of EPS–gelatin films make them a promising candidate for biodegradable coatings and biomedical applications, including wound dressings and drug-delivery materials.

## Conclusion

This study investigated the impact of microbial mutagenesis on EPS productivity and properties in *Leu. mesenteroides* WiKim33 strains. The ALE1 strain exhibited the highest EPS productivity, while the ALE2 strain had the lowest. 1D and 2D NMR spectra confirmed that genetic modifications did not alter the chemical backbone of the EPS. For physicochemical characterization of EPSs, SEC-MALS was applied to measure molecular weight, and DLS was used to evaluate hydrodynamic size. DLS measurement revealed significant differences in the hydrodynamic size of the EPS, with ALE1 producing the smallest molecules. The study further revealed that smaller EPS decreased water solubility through decreased phase separation due to homogeneous film formation. The properties of EPS films could be effectively altered to the different hydrodynamic size of EPS produced by mutant strains. These findings highlight the potential of EPS from mutagenized strains for specialized biomaterials such as food packaging, pharmaceuticals, and biodegradable films. Future research should focus on optimizing the production and functionalization of EPS-based materials to further explore their industrial and environmental applications.

## Glossary

Adaptive Laboratory Evolution (ALE):

A process in which microorganisms undergo repeated stress in a controlled laboratory setting to induce adaptive genetic changes, enhancing their stress tolerance.

Confocal Laser-Scanning Microscopy (CLSM):

A microscopic imaging technique that uses laser scanning to create high-resolution images of biofilms or polymer matrices, enabling the analysis of spatial distributions and structural details.

Differentially Expressed Genes (DEGs):

Genes that exhibit significant expression differences under various conditions, often as part of an organism's adaptive response to environmental stress.

Dynamic Light Scattering (DLS):

A technique for measuring the size distribution of molecules in solution by analyzing light scattered from their Brownian motion.

Exopolysaccharides (EPS):

Polysaccharides secreted by microorganisms outside their cells, essential for biofilm formation and protective functions.

Heteropolysaccharides (HePS):

Polysaccharides composed of different monosaccharides, which provide structural diversity and functional variety.

Homopolysaccharides (HoPS):

Polysaccharides consisting of a single type of monosaccharide, typically resulting in a more uniform structure. Lactic Acid Bacteria (LAB):

A group of bacteria known for producing lactic acid during carbohydrate fermentation; commonly used in food fermentation and probiotics.

Nuclear Magnetic Resonance (NMR):

A spectroscopic technique that reveals detailed information about molecular structures by analyzing the magnetic properties of atomic nuclei.

Single Nucleotide Polymorphism (SNP):

A variation at a single nucleotide in the DNA sequence across different individuals or strains, contributing to genetic diversity and adaptation.

Two-Dimensional Nuclear Magnetic Resonance (2D NMR):

An advanced NMR technique, including COSY and HSQC, used to determine spatial and scalar correlations between protons and carbons in complex molecules.

Wild Type (WT):

The natural, non-mutated strain of an organism, often used as a baseline or control in studies comparing mutated or evolved strains.

## Supplemental Materials

Supplementary data for this paper are available on-line only at http://jmb.or.kr.



## Figures and Tables

**Table 1:** Genetic modification of *Leuconostoc mesenteroides* WiKim33 ALE1 and ALE2 during heterotypic shock induced evolutionary engineering.

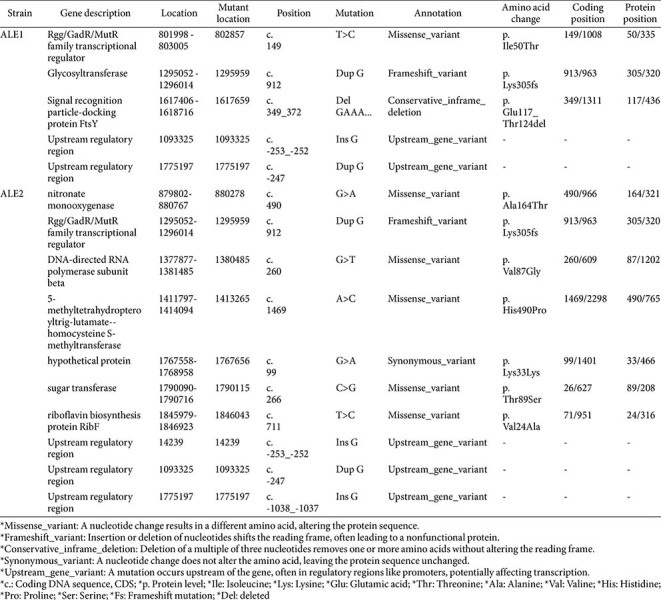

**Table 2:** EPS productivity of *Leuconostoc mesenteroides* WIKim33 WT and mutants in MRS media.

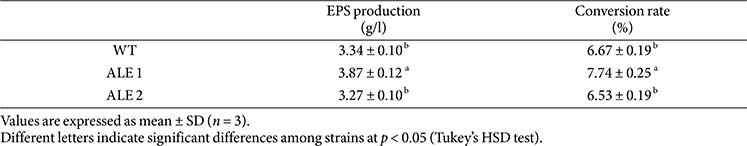

**Table 3:** Comparing properties of EPS films.