# Supplementary material for: Hydrodynamic Size Modulation of Exopolysaccharides from Leuconostoc mesenteroides WiKim33 via Genetic Modification and Its Impact on the Properties in Film
Source: J Microbiol Biotechnol. 2025 Nov 27;35:e2508008. doi: 10.4014/jmb.2508.08008 (PMC12685584; doi:10.4014/jmb.2508.08008)
Supplement: Supplementary file 1 [file jmb-35-e2508008-supple.pdf]

J. Microbiol. Biotechnol. 2025. 35: e2508008

https://doi.org/10.4014/jmb.2508.08008

Supplementary Data

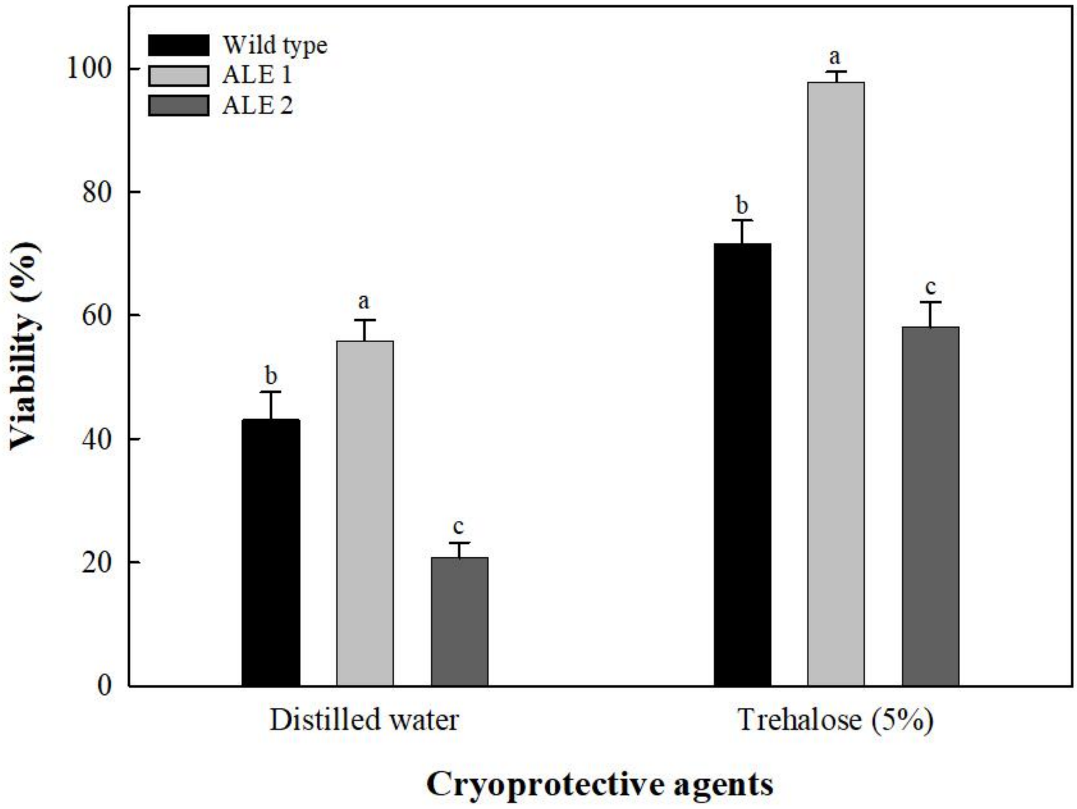

Supplementary Data 1. Freeze-drying tolerance of *Leuconostoc mesenteroides* WiKim33 and mutants

Supplementary Data 2. Mutant gene descriptions of *Leuconostoc mesenteroides* WiKim33 during heterotypic shock induced evolutionary engineering.

Gene	Description	References
Rgg/GadR/MutR family transcriptional regulator	Controlling various physiological events such as oxidative stress responses, non-glucose sugar metabolism, bacteriocin production, quorum sensing, virulence, and biofilm formation	[28-31]
Glycosyltransferase	Transferring sugar moieties to various substrates.<br>Effect on the bacterial glycosylation process, such as the synthesis of glycolipid, glycoproteins, and polysaccharides<br>Structural differences in glycosyltransferase can influence enzymatic activity, donor sugar binding, and acceptor substrate binding sites	[32]
Signal recognition particle-docking protein FtsY	Involving targeting and insertion of nascent membrane proteins into the cytoplasmic membrane<br>Binding to these sites in stabilized by blocking its GTPase activity	[33]
Nitronate monooxygenase (NMO)	Playing an important role in bacterial defense mechanisms<br>Playing a role in conserving motif and oxidizing propionate-3-nitronate	[34]
Rgg/GadR/MutR family transcriptional regulator	-	-
DNA-directed RNA polymerase subunit beta (rpoB)	Playing a role in capsular synthesis, branched-chain amino acid synthesis, ABC transporters/oligopeptide & nickel transport, regulation	[35]
5-methyltetrahydropteroyltriglutamate--homocysteine S-methyltransferase (MetE)	The final step of methionine biosynthesis<br>Playing an important role in mediating environmental stress through the calmodulin signaling pathway	[36]
Hypothetical protein	-	-
Sugar transferase	Playing a role in biosynthesis bacterial capsular polysaccharides, have been highlighted as sources of glycol engineering	[37]

Supplementary Data 3. The assignments of ^{13}C NMR chemical shifts for EPSs.

Carbon atom	Current study			*Leuconostoc mesenteroides* KIBGE-IB22M20 [18]	*Leuconostoc mesenteroides* CMG713 [38]	*Leuconostoc mesenteroides* NRRL B512F [39]
	WT	ALE1	ALE2			
C-1	97.73	97.73	97.73	97.2	100.56	98.53
C-2	71.42	71.42	71.41	71.1	74.25	72.23
C-3	73.41	73.42	73.41	73.2	76.25	74.23
C-4	69.55	69.55	69.55	70.0	73.04	71.03
C-5	70.20	70.20	70.20	69.8	72.43	70.35
C-6	65.57	65.57	65.57	66.8	68.48	66.35

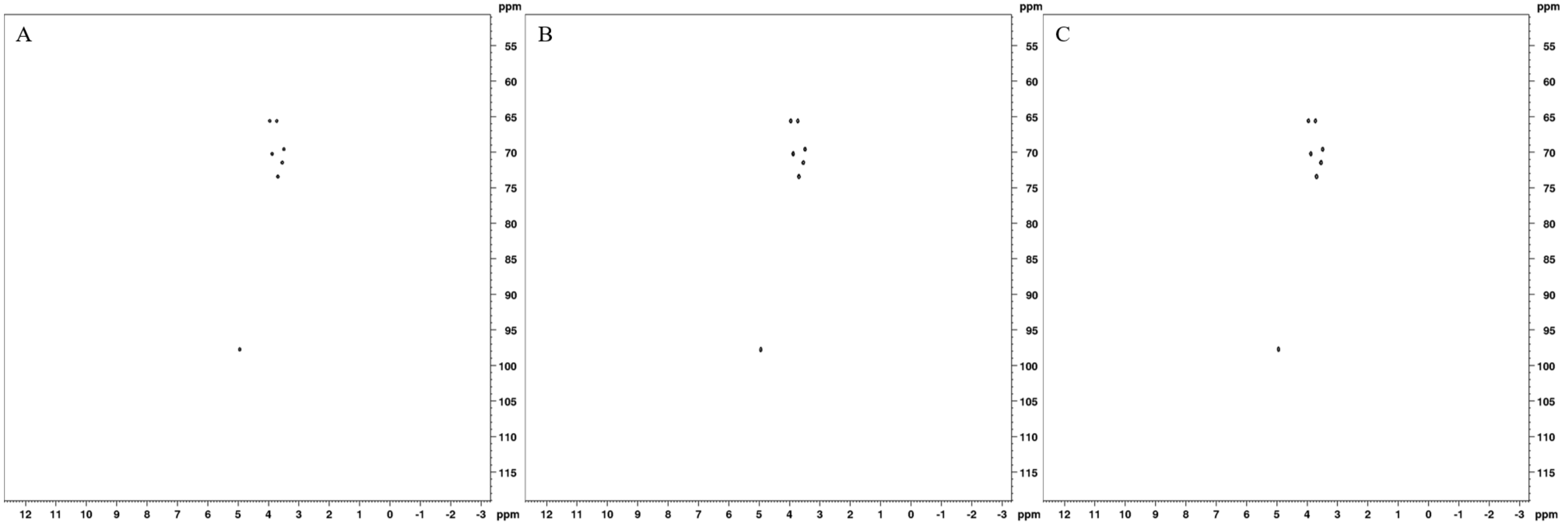

Supplementary Data 4. 2D HSQC NMR full spectra of EPSs purified from WT, ALE1, and ALE2 waste media. (A-C) NMR spectra of EPSs purified from WT, ALE1, and ALE2 waste media, respectively.

Supplementary Data 5. Molecular weight analysis of EPSs purified from WT, ALE1, and ALE2 waste media.

	Mn (×10^6 g/mol)	Mw (×10^6 g/mol)	Mz (×10^6 g/mol)	PDI (Mw/Mn)
WT-EPS	6.46	7.06	7.54	1.09
ALE 1-EPS	6.37	7.01	7.55	1.10
ALE 2-EPS	6.29	7.01	7.59	1.12

Different letters above bars indicate values that are significantly different at $P < 0.05$ (Tukey's honestly significant difference test).

Supplementary_data_6..mp4

Supplementary Data 6. Three-dimensional confocal microscopy observation of EPS film.
